# Comparative evaluation of internal fit and marginal gap of endocrowns using lithium disilicate and polyether ether ketone materials - an in vitro study

**DOI:** 10.1186/s12903-023-02857-8

**Published:** 2023-04-07

**Authors:** Nermeen Nagi, Ahmed Mahmoud Fouda, Christoph Bourauel

**Affiliations:** 1 Bonn University, Bonn, Germany; 2 Faculty of Dentistry, Galala University, Suez, Egypt; 3 Faculty of Dentistry, Fayoum University, Fayoum, Egypt; 4 Oral Technology, University Hospital Bonn, Bonn, Germany

**Keywords:** Endocrowns - Lithium disilicates, PEEK, Internal fit, Marginal gap

## Abstract

**Background:**

The aim of the study was to investigate the effect of material and occlusal preparation design on the internal fit and marginal gap of endocrowns made of Polyether ether ketone (PEEK) and lithium disilicate.

**Methods:**

32 endocrowns were fabricated on prepared mandibular molars and divided into two groups (n = 16) according to the material. Group L: lithium disilicate and Group P: PEEK. Each group was further subdivided into two subgroups (n = 8) according to the occlusal preparation design: full occlusal coverage (LF and PF) and partial occlusal coverage (LP and PP). Samples were analyzed using microcomputed tomography (µCT) with a voxel size of 6 μm to evaluate internal fit, and an optical microscope was used to evaluate the marginal gap. Data were collected, tabulated, and statistically analyzed. Numerical data were described as mean and standard deviation and compared using the ANOVA test. The level of significance was set at α P ≤ 0.05.

**Results:**

All groups’ internal fit and marginal gaps values were within the acceptable clinical range. However, the lithium disilicates group recorded statistically significantly higher mean internal gap values than the PEEK groups. Regardless of the material, the difference between the two occlusal designs was not statistically significant in both internal fit and marginal gap records.

**Conclusion:**

Within the limitations of this study, PEEK endocrown restorations revealed better internal fit and marginal gap than lithium disilicate endocrown restorations. The marginal and internal fit of both lithium disilicate and PEEK endocrown restorations were within the clinically acceptable range. The occlusal preparation design had no influence on the internal fit and marginal gap of the endocrown restoration.

## Background

The increasing demands for conservatism seeking the preservation of sound tooth structure and the innovation in dental materials in the last years led to searching for another approach to restoring endodontically treated teeth. Using endocrowns has become a good option for restoring endodontically treated teeth with the current adhesion protocols [1]. Glass ceramics (Feldspathic, Leucite, or lithium (di) silicate-based), reinforced glass ceramics with Zirconia or hybrid resin nanoceramics were materials of choice for fabrication of endocrowns because of their outstanding esthetics, low thermal conductivity, adequate strength, biocompatibility, wear resistance, and chemical stability [2]. Additionally, these materials are etchable and have the ability to adhesively bond to tooth structure due to their silica content. The silica is selectively removed by the hydrofluoric acid etchant, creating topographic changes that enhance micromechanical retention and chemical bonding to resin cement [3]. On the other hand, the inherited brittleness of etchable ceramics was claimed to be the major limitation of these materials as it leads to catastrophic fracture and excessive wear on opposing natural teeth. To overcome this problem, using a material with the potential for stress distribution as polyether ether ketone **(**PEEK) has been suggested.

PEEK is a polyaromatic semi-crystalline thermoplastic polymer introduced in the early 1980s, mainly for engineering applications such as aircraft manufacturing, piston parts, turbine blades, and cable insulation [4]. Later in 1998, it was introduced for biomedical applications. Victrex PLC (Imperial Chemical Industry, London, UK) produced PEEK-OPTIMA for long-term implant applications [5].

PEEK is widely used in dentistry because of its excellent thermal properties, high wear resistance, good processability, corrosion resistance, relatively high modulus of elasticity, and low density (1.32 g/cm^3^) [6]. Moreover, the tensile properties of PEEK are comparable to enamel and dentin [7]. It also has no metallic color; instead, it is beige with a touch of grey, making it a suitable restorative dental material [8]. Because of the mentioned properties and the excellent biocompatibility of PEEK, it is successfully used in dentistry as implants, abutments, a framework for removable prosthesis, and fixed restorations [7]. The inertness and poorly adhesive hydrophobic surface (surface contact angle, θ at 65°) of PEEK is an essential obstacle to its wider application in fixed prosthodontics [9]. So, different surface treatments have been used to improve the bonding of PEEK with various luting cements [7].

Despite the significant role of modern adhesives in improving the retention and mechanical properties of endocrowns, it is not the only factor that affects the retention of the restoration. The importance of marginal and internal gap width of endocrowns has been emphasized in several clinical trials [10]. Poor marginal adaptation may lead to the gradual degradation of the luting cement’s chemical, physical, and mechanical properties. That will result in microleakage, recurrent caries, discoloration of the tooth structure, and restoration failure [11]. Furthermore, internal fit influences the cement thickness which is crucial to the mechanical stability of adhesive cement layer at the tooth/restoration interface [12].

In order to expand the clinical indications of PEEK as an endocrown, it is important to understand the material’s behavior and compare it to the widely used lithium disilicate endocrowns. The null hypothesis is that PEEK and Lithium disilicate endocrowns will have a similar internal fit and marginal gaps regardless of the preparation design.

## Methods

### Sample size calculation

Statistical power analysis was carried out to determine the sample size using G*power (version 3.1.9.4, Heinrich-Heine-Universität Düsseldorf) based on previously published results [13, 14]. A sample size of 8 samples per group was sufficient to detect a large effect size (f) ranging from 0.657 to 0.669, with an actual power (1-β error) of 0.8 (80%) and a significance level (α error) of 0.05 (5%) for a two-sided hypothesis test.

### Study design

thirty-two endocrowns were fabricated and divided into two main groups according to the material (n = 16): group L (lithium disilicate) and group P (PEEK). Each group was further subdivided into two subgroups according to the occlusal preparation design (n = 8). Full occlusal coverage (denoted by letter F), and partial occlusal coverage (denoted by letter P).

### Teeth preparation

Thirty-two extracted mandibular molars of nearly similar size were collected from a pool of anonymized teeth obtained from the surgery department at the university hospital after approval from the ethics committee to carry out the study. The teeth entered the pool after written and signed informed consent. Mesiodistal and buccolingual dimensions of the teeth were measured at the cementoenamel junction (CEJ), and a maximum deviation of 10% was allowed. The inclusion criteria were absence of caries, old fillings or cracks at 2x magnification, and complete root formation. The teeth were disinfected in 0.2% Thymol solution for 48 h and stored in normal saline.

**Fig. 1:**
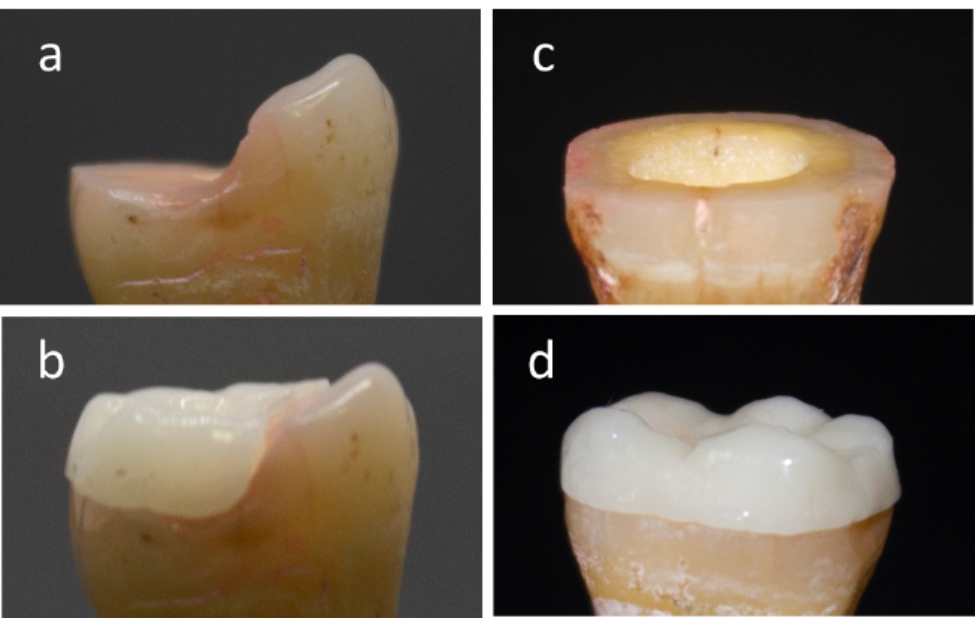
(a) partial coverage endocrown preparation, (b) partial coverage endocrown restoration, (c) full coverage endocrown preparation, (d) full coverage endocrown restoration

Teeth were prepared with an access cavity using a diamond bur then root canals were prepared using a sequence of files (K-Flex) according to the manufacturer’s instructions. Irrigation was performed using a 5.25% sodium hypoclorite solution for 2 min [15]. Teeth were filled with gutta-percha cones. Under constant water irrigation, an 8-degree tapered diamond rotary instrument (#856; Intensiv SA, Montagnola, Switzerland) was used to perform intra-coronal preparation at 2.5x magnification. The excessive retentive areas were removed, the pulpal walls were aligned, and internal angles were rounded, resulting in 2 mm thickness axial walls. The preparations were smoothed using a fine polishing rotary instrument (#504; Intensiv SA, Montagnola, Switzerland)). Half of the teeth were prepared by removing the coronal portion of the tooth horizontally, leaving 2 mm above the cementoenamel junction (CEJ), forming a butt margin preparation design, while the other half was prepared by removing only the buccal cusps leaving 2 mm intact tooth structure cervically and leaving the lingual cusps unprepared (Fig. 1). To standardize the depth of the pulp chamber, composite resin was added as base material using incremental packing technique. All preparations were performed by a single operator.

### Endocrowns fabrication

The prepared teeth were scanned using Medit I500 intraoral scanner (MEDIT corp., Seoul, Republic of Korea). Endocrown restorations were designed using DentalCAD 3.0 Galway Exocad software (exocad GmbH, Darmstadt, Germany). The cement spacer was set at 50 µm thickness, and the marginal adhesive gap was set at 0 µm for all specimens. Sixteen monolithic endocrowns, 8 for each preparation design, were milled out of lithium disilicate glass-ceramic blocks (IPS e.max CAD blocks, Ivoclar Vivadent, Schaan, Liechtenstein). Another 16 endocrowns, 8 for each preparation design, were milled from Ceramill PEEK blanks (Amann Girrbach AG, Koblach, Austria). The restorations were made by CEREC In Lab MCXL milling unit with milling burs size 0.6mm (Dentsply Sirona, North Carolina, U.S.A). All the endocrowns were then finished according to the manufacturer’s guidelines. The teeth were selectively etched using Scotchbond Universal 37% phosphoric acid gel (3 M, Maplewood, Minnesota, USA). The etching gel was applied for 30 seconds on enamel and 15 seconds on dentin then the teeth were thoroughly rinsed. Tetric N-Bond Universal (Ivoclar Vivadent, Liechtenstein) was applied and light cured for 20 sec according to the manufacturer’s instructions using SmartLite Pro light curing unit (Dentsply Sirona, North Carolina, U.S.A). All the endocrowns were finished and treated according to their manufacturer’s guidelines. Surface treatment of lithium disilicate restorations was done by Prime-Dent porcelain etch gel 10% hydrofluoric acid (Prime Dental, Chicago, U.S.A) for 60 seconds, followed by rinsing. Afterward, Silane-it (ITENA, Villepinte, France) silane coupling agent was applied to the fitting surface of restorations [16]. The surface treatment of PEEK restorations was done by application of 98% sulfuric acid etching on the fitting surface for 30 seconds, followed by rinsing. Breeze Automix Self-Adhesive dual-cured opaque resin cement (Pentron, Wallingford, United States) was applied to the teeth’ internal surface and restorations [17]. Each endocrown was seated, and 2–4 s tuck curing was done according to the manufacturer’s instructions to facilitate the removal of excess cement. And then held in position by exerting constant finger pressure for 5 min [18].

### Internal fit analysis

**Fig. 2:**
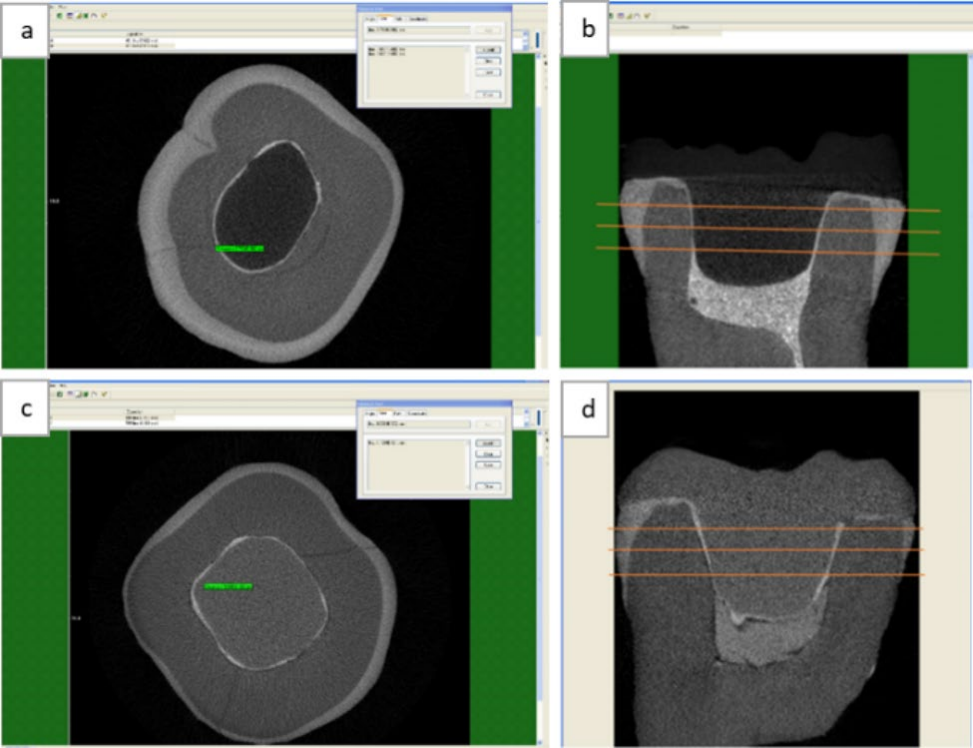
µCT Scan analyzer measurements of cement line thickness a: Cross-sectional slice with cement line measurements in PEEK endocrown, b: Longitudinal section showing levels of cross-sectional slices in PEEK restoration, c: Cross-sectional slice with cement line measurements in lithium disilicate endocrown, d: Longitudinal section showing levels of cross-sectional slices in lithium disilicate endocrown

Samples were embedded in cylindrical PVC rings 2 mm away from the CEJ using auto-polymerized colorless acrylic resin. To allow positioning of all samples at equal distances from the center of rotation of the micro-computed tomography scanner (Skyscan 1174™, Skyscan, Kontich, Belgium), thus providing a standardized image quality. Acquisition of µCT images was made using the same parameters for all samples. Imaging was performed at a high resolution (6 μm voxel size) using a tube voltage of 50.0 kV and a tube current of 800 µA, 9500 ms integration time, 180° rotation angle, and 0.4° angular step. A 1 mm Aluminum filter filtered the x-ray spectrum. Reconstruction was done for all scans. Measurements were taken using Scan Analyzer software (Rohmann GmbH, Frankenthal, Germany). Each sample was measured in three different levels of cross-sectional slices. Three readings were taken for the cement line thickness from each surface (buccal, lingual, mesial, and distal). (Fig. 2)

### Marginal gap analysis

Stereomicroscope (Wild M8, Heerbrugg, Switzerland) with a magnification of 50x was used to evaluate the interfaces between the restoration and the tooth structure to measure the marginal gap filled with residual luting cement. This gap (i.e., the dental cement thickness/width) has been defined as the maximum distance between the underlying prepared tooth’s finish line and the restoration’s margin. This width of the marginal residual luting cement must be studied because it is related to the microleakage potential [19].

**Fig. 3:**
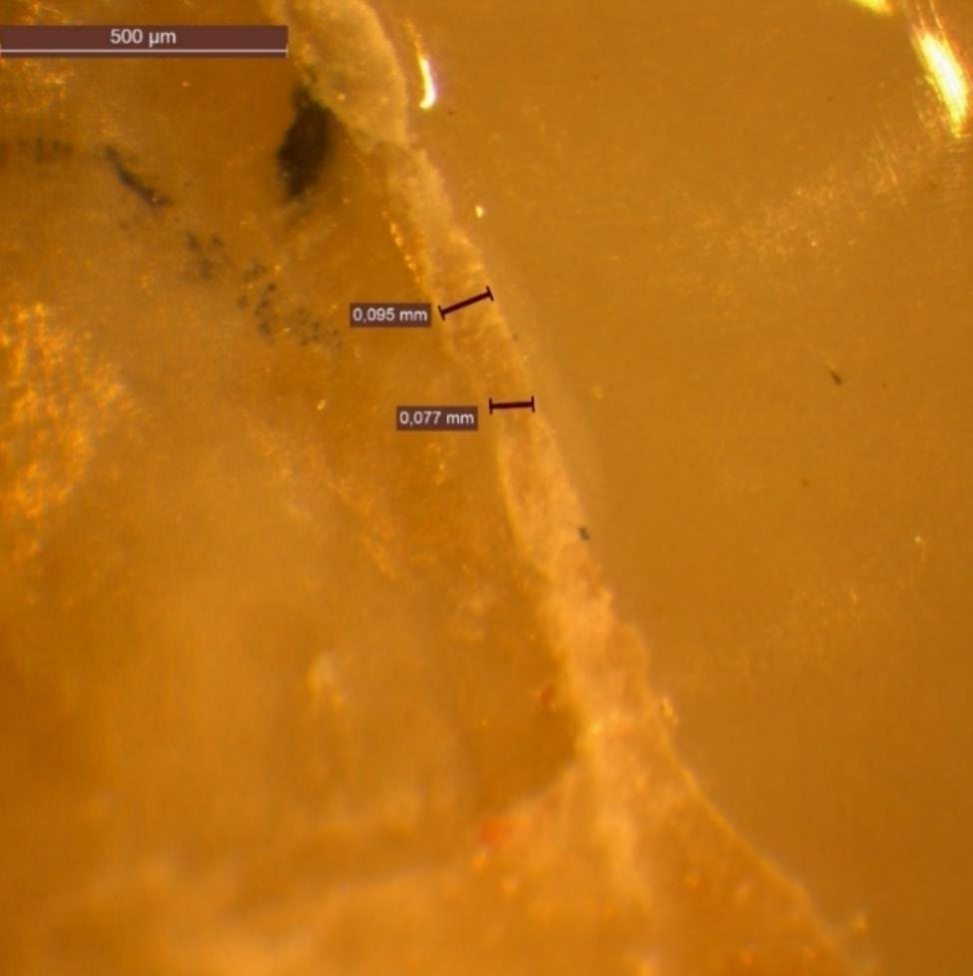
Points of marginal gap measurements between prepared tooth and lithium disilicate endocrown under the stereomicroscope

Four surfaces were considered for each tooth: buccal, lingual, mesial, and distal. Each corresponding interface was divided into three segments, thus generating the following sites of interest: buccal surface: (mesial third, middle third, and distal third). Lingual surface: (mesial third, middle third, and distal third) Thus, 6 points are considered for each surface (Fig. 3), while mesial surface and distal surface measurement were considered only in the middle segment of the surface. That was because of these surfaces’ convexity, making it difficult to gain focused points at the periphery. The marginal gaps were measured in two points for each segment. Thus, 16 points of measurement are considered in total along the adhesive interfaces for each tooth.

All data were collected, tabulated, and statistically analyzed. The data were saved as a hard and a soft copy and stored in the database of the Oral Technology Department, University Hospital Bonn.

### Statistical analysis

Data were explored for normality by checking the data distribution using Kolmogorov-Smirnov and Shapiro-Wilk tests. Data management and statistical analysis were performed using SPSS software (V.18, IBM Inc., New York, USA). Numerical data were summarized using mean, and standard deviation.

Comparisons between groups were performed using the ANOVA test, and Bonferroni post hoc test for pairwise comparison. Qualitative data (failure) were compared using chi square test. All p-values are two-sided. P-values ≤ 0.05 were considered significant.

## Results

Internal fit analysis showed that the highest mean internal gap value was recorded in the LF group (127 ± 14 μm), while the least value was recorded in the PP group (100 ± 8 μm). In full coverage occlusal design restorations PEEK showed a significantly lower mean internal gap (104 ± 19 μm) than lithium disilicates (127 ± 14 μm). On the other hand, there was no significant difference in the mean internal gap values between PEEK (100 ± 8 μm) and lithium disilicate (112 ± 10 μm) with partial coverage occlusal design.

Marginal gap analysis results demonstrated that the LF group had the highest mean marginal gap (109 ± 14 μm), while the PP group had the lowest one (64 ± 11 μm). Regardless of the design, PEEK showed significantly lower mean marginal gap values than lithium disilicate.

Although partial coverage occlusal design specimens had slightly lower internal and marginal gaps, the restoration design did not significantly affect the internal fit or marginal fit of PEEK or lithium disilicate endocrowns. (Table 1)

**Table 1:** Descriptive statistics and comparison of marginal gap and internal fit (µm)

		Mean	95% Confidence Interval for Mean		Min	Max	F	P
			Lower Bound	Upper Bound				
Internal fit	PF	104 ± 19a	91	121	92	148	6.036	< 0.001
	PP	100 ± 8a	93	108	86	117		
	LF	127 ± 14b	116	139	104	147		
	LP	112 ± 10ab	104	121	97	128		
Marginal gap	PF	87 ± 23ab	67	107	62	134	11.450	< 0.001
	PP	64 ± 11b	55	74	45	82		
	LF	109 ± 14c	98	122	90	134		
	LP	98 ± 11ac	89	109	85	117		

* Means that share a letter are not significantly different

## Discussion

Lithium disilicate is still widely used to fabricate endocrowns. However, ceramics have drawbacks that might impair the result of endocrowns, including greater stiffness and rigidity, which could compromise marginal adaptation [20]. In 2016, Zoidis et al. proposed that PEEK can be used for endocrown restorations as it can withstand the physiologic occlusal forces successfully [21]. The modulus of elasticity of PEEK is up to 4 GPa, close to that of the human cortical bone (15–20 GPa). Furthermore, it is very rigid, with a flexural strength of 140–170 MPa [22]. These properties help to dampen the occlusal forces protecting the weak tooth structures better than the brittle ceramic materials [23, 24].

The clinical success and lifetime of dental restoration depend significantly on the restoration’s marginal adaptation and internal fit. Increased marginal and internal gaps will increase the thickness of the luting cement layer, which dissolves in the oral environment and negatively affects the restorations’ longevity and success [25].

Endodontically treated molar teeth were selected in the current study because of the high success rate of endocrown restorations in molars. They have a large pulp chamber, more surface area for bonding, and better stress distribution [26].

Choosing butt joint marginal preparation in the full coverage design was to minimize the complexity of the design and to get less marginal and internal discrepancies. On the other hand, the Partial coverage preparation design was suggested to be more conservative and used with teeth with intact buccal cusps to preserve the remaining tooth structure as much as possible [27].

Prepared teeth received either monolithic lithium disilicate restorations in the posterior region, where monolithic restorations are esthetically accepted or PEEK restorations made of beige shaded blocks, as this shade was esthetically accepted. As those restorations were in a high-stress posterior zone, no veneering material was added to eliminate the risk of chipping [28].

According to the literature endocrowns made of lithium disilicate ceramics are considered among the best restorative materials due to their good adhesive properties and micromechanical interlocking with resin cements [29]. Gudugunta et al. and Dolev et al. compared the marginal fit between hot-press and CAD-CAM lithium disilicate crowns. They concluded that the marginal fit of crowns fabricated by the CAD-CAM technique was better than the pressed technique [30]. Therefore, in the current study, CAD-CAM technique of fabrication was employed. The restorations were designed on Exocad software which provides easy and proper designing of the restorations with its large built-in library with different outlines to choose from [31].

A 5-axis milling machine was used to fabricate the restoration in the current study because the type of milling device could affect the adaptation of the restoration, especially if it has a complex shape, deep groove regions, and internal angles [32]. When different milling units were compared best accuracy of fit was recorded with 5-axis milling machines [33]. Also, the bur size and shape of the milling unit can influence the adaptation of a restoration. A small diameter of 0.6 mm should be used when milling complex shapes [34].

Acid-etched (98% sulfuric acid) PEEK surfaces provide a good shear bond strength with the adhesive resin cements and therefore have favorable bonding properties [35].

Lithium disilicate and PEEK endocrowns were cemented using dual-cure self-adhesive resin cement to reach optimum bond strength with dentin. Dual-cured resin cement was chosen as the thickness of some areas might be more than 2 mm, ensuring complete polymerization [36].

Using 2D analysis to evaluate the internal fit of the restoration provides a limited number of points that can be measured. Therefore, results may not represent the actual fit of the restoration [37]. Therefore, 3D microcomputed tomography was used to evaluate the internal fit of the restoration [38, 39]. This technique has high validity and reliability as it provides more point measurements that cannot be achieved with a 2D technique [40, 41]. The marginal fit of the restoration was evaluated with a stereomicroscope as Stereomicroscopy at a value less than or equal to 30 μm was used as a gold standard to evaluate the significance of different designs on marginal adaptation [19].

The results of the current study have shown that CAD-CAM endocrowns fabricated with PEEK have better marginal and internal fit compared to lithium disilicate endocrowns. However, the marginal gaps of both CAD-CAM materials were within the clinically acceptable range of 109 ± 14 μm, and the internal gap was in the range of ≤ 127 ± 14 μm [42]. The values are less than those observed by Shin et al., Who reported internal discrepancies for CAD-CAM endocrowns of 200–300 μm [43]. This difference could be attributed to the fact that the internal fit of endocrowns is influenced by the cavity depth, restoration material and processing techniques [44].

Also, the results agreed with a study by Osman, A. M et al. (2022) [45] in which E-Max restorations recorded higher internal and marginal gap mean values than PEEK restorations. Makky, (2020) [46] explained the smaller marginal gap of PEEK because polymer-based materials have better adaptation than brittle glass-ceramics as there is no marginal chipping during the finishing of PEEK .

All restorations in the current study meet the requirements regarding a clinically acceptable marginal and internal gap, irrespective of the preparation design used. Although there was no statistically significant difference between the occlusal preparation designs, the partial coverage occlusal design of endocrowns showed better internal fit and less marginal gap readings than the full coverage occlusal design, which was in accordance with the findings of Merrill et al. (2021) [47]. This could be due to the influence of the design on the flowing off of the luting material during the cementation process. The factors responsible for these findings require further substantiation [48].

In our study, the preparation design did not influence internal fit and marginal gap. Nevertheless, as reported by other studies, cyclic fatigue tests have outlined significant differences after cyclic load [49]. Further studies that include cyclic fatigue are needed to better evaluate the influence of the type of material and the preparation design on the internal fit and marginal gap.

The promising properties of PEEK make it a sought-after material for endocrown restorations. Since this study was performed under in-vitro conditions, further long-term randomized clinical trials on a larger population are recommended to confirm the results of this study. Also, further studies are required to evaluate other criteria not covered in this study (anatomic shape, surface roughness, restoration staining, color match, and patient satisfaction).

## Conclusion

Within the limitations of this study, the following conclusions could be drawn:

PEEK revealed better internal fit and lowered marginal gap than lithium disilicate endocrown restorations.

The marginal and internal fit for lithium disilicate and PEEK endocrown restorations were within the clinically acceptable range.

The occlusal preparation design does not influence the internal fit and marginal gap of the endocrown restoration.

## Data Availability

All data and results analyzed during the current study are available from the corresponding author upon reasonable request. Moreover, they have stored as hard and soft copies in the database of the Oral Technology Department, University Hospital Bonn.

## Abbreviations

PEEK: Polyether ether ketone
µCT: Micro-computed tomography
CEJ: Cementoenamel junction
